# Genetic susceptibility to advanced retinopathy of prematurity (ROP)

**DOI:** 10.1186/1423-0127-17-69

**Published:** 2010-08-25

**Authors:** Barkur S Shastry

**Affiliations:** 1 Department of Biological Sciences, Oakland University, Rochester, MI, USA

## Abstract

Retinopathy of prematurity (ROP) is a vascular vitreoretinopathy that affects infants with short gestational age and low birth-weight. The condition is a multifactorial disease and is clinically similar to familial exudative vitreoretinopathy (FEVR), which is a bilateral hereditary eye disorder affecting full-term infants. Both of them are characterized by the abnormal vessel growth in the vitreous that can lead to vitreoretinal traction, retinal detachment and other complications resulting in blindness. Despite the recent advances in diagnosis and treatment, ROP remains a major cause of childhood blindness in developed countries. The etiology of pathogenesis of advanced ROP is currently unknown. In the past, many causative factors such as length of time exposed to supplemental oxygen, excessive ambient light exposure and hypoxia have been suggested but evidence for these as independent risk factors in recent years is not compelling. It is not clear why ROP in a subset of infants with low birth-weight progresses to a severe stage (retinal detachment) despite timely intervention whereas in other infants with similar clinical characteristics ROP regresses spontaneously. Recent research with candidate gene approach, higher concordance rate in monozygotic twins and other clinical and experimental animal studies, suggest a strong genetic predisposition to ROP besides environmental factors such as prematurity. Three genes, which are involved in the Wnt signaling pathway, are mutated in both FEVR and in a small percentage of ROP disorder. However, none of the genetic factors identified thus far in ROP, account for a substantial number of patient population. Future studies involving genomics, bioinformatics and proteomics may provide a better understanding of the pathophysiology and management of ROP.

## Introduction

**Figure 1:**
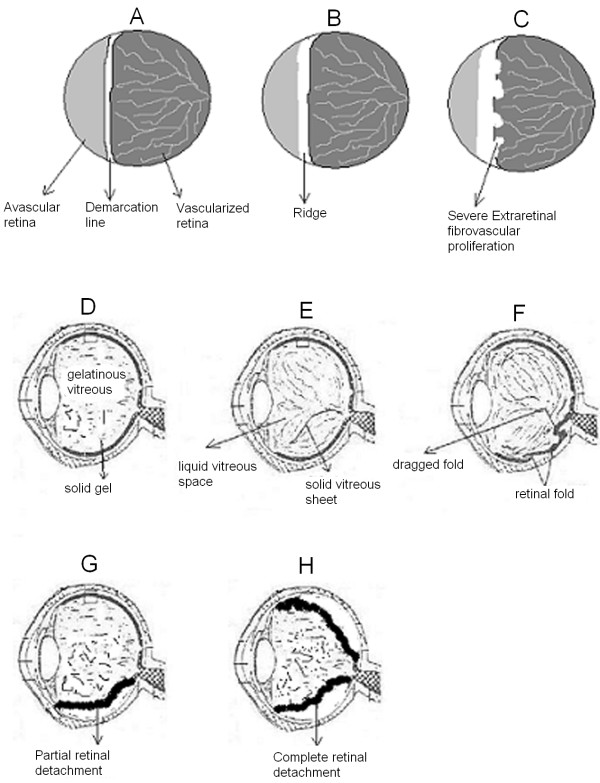
**A schematic illustration of some of the abnormalities associated with advanced ROP**. In the early stages, ROP is characterized by an incomplete vascularization of the peripheral retina (panel A), with a sharply demarcated boundary between vascularized and avascularized retina (stage 1). This can progress to an elevated ridge (panel B) that consists of mesenchymal tissue (stage 2). In more advanced stages of the disease, extra-retinal fibrovascular proliferation occurs (panel C) on the posterior border of the ridge (stage 3). In addition to the abnormal vascularization, the normal gelatinous vitreous (panel D) becomes partially liquefied (panel E). While spontaneous regression often occurs, an organization and contraction of vitreous collagen can take place (panel E) which can lead to a retinal fold (panel F) causing partial (panel G) or total retinal detachment (panel H) that represent stages 4 and 5 respectively.

Retinopathy of prematurity (ROP), also known as retrolental fibroplasia, is a well-known visual impairment in premature children. It is a disease of developing retinal blood vessels, which is seen predominantly in infants of short gestational age and low birth-weight. The condition is a multifactorial disease and is clinically similar to familial exudative vitreoretinopathy (FEVR), which is a bilateral hereditary eye disorder affecting full-term infant. While FEVR is a genetically heterogeneous disorder and is inherited as an X-linked recessive, autosomal dominant (AD) and autosomal recessive (AR) mode [1], ROP is a non-familial disorder. Both of these disorders cause blindness in young children and are characterized in the early stage by the premature arrest of the vascularization of the peripheral retina (Fig. 1) that can lead to extra-retinal fibrovascular proliferation, vitreal traction, retinal fold and retinal detachment resulting in blindness. Both of them carry a high financial cost for the community and the individual by affecting the normal motor, language, conceptual and social development of the child. It is estimated that the overall incidence rate of the condition is about 68% among infants born with birth weight less than 1251 g and 98% among children born with birth weight less than 750 g [2].

## Pathogenesis

The International Classification of ROP divides the development of the disorder into 5 stages. In the early stages, ROP is characterized by an incomplete vascularization of the retina (Fig. 1 panel A), with a sharply demarcated boundary between vascularized and avascularized retina (stage 1). This can progress to an elevated ridge (Fig. 1 panel B) that consists of mesenchymal tissue (stage 2). In more advanced stages of the disease, extra-retinal fibrovascular proliferation occurs (Fig. 1 panel C) on the posterior border of the ridge (stage 3) and may be associated with dilated and tortuous retinal vessels (stage 3+ or threshold). In addition to the abnormal vascularization, the normal gelatinous vitreous (Fig. 1 panel D) becomes partially liquefied (Fig. 1 panel E). While spontaneous regression often occurs, an organization and contraction of vitreous collagen can take place (Fig. 1 panels D, E and F) leading to partial (stage 4) or total (stage 5) retinal detachment (Fig. 1 panels G and H respectively). In advanced ROP, abnormal vessels grow out of the retina into the vitreous. This abnormal growth can lead to hemorrhage, fibrovascular changes, vitreoretinal traction, and retinal detachment (Fig. 1) and ultimately results in blindness.

Additionally, other complications of ROP or current treatment have also been reported. This includes retinal fold, dragging of the macula, glaucoma, cataract and strabismus. It is a life time disease. The condition in some children may appear milder and may not require treatment during the active stages or it may be regressed with little or no loss of visual function. However, these same children may later develop visual impairments from progressive retinal epithelial changes [3,4]. This type of childhood blindness has severe consequences that may result in less opportunity for education, employment and earning potential. It can also affect socioeconomic development.

## Genetic risk factors

Despite advances in our understanding and management of ROP, it remains a leading cause of blindness in children in developed countries. Although many causative factors such as excessive light exposure, length of time exposed to supplemental oxygen and hypoxia have been suggested [5,6], the etiology of pathogenesis of advanced ROP is not understood. However, low birth-weight and short gestational age have been consistently shown to be associated with ROP. It is unclear why ROP in a subset of infants with low birth-weight progresses to a severe stage despite timely intervention whereas in other infants with similar clinical characteristics, ROP regresses spontaneously. Molecular genetic studies of FEVR have identified four causative genes to date (NDP, FZD4, LRP5 and TSPAN12) which when mutated cause X-linked, AD and AR FEVR (also some sporadic cases). All of these genes are involved in the beta-catenin mediated Wnt signaling pathway (see below) that controls the development of the retinal vasculature [7-9]. Because of mutations in these genes, norrin-FZD4-LRP5-TSPAN12 signaling pathway may become defective and that may produce abnormal vascularization giving rise to FEVR pathology. Interestingly, using a candidate gene approach, it has been shown that at least three of the four FEVR genes (NDP, FZD4 and LRP5) are also mutated in a small percentage (3-11%) of severe ROP patients [[10-19] and Hiraoka etal. personal communication]. This genetic predisposition is further supported by the recent twin studies [20], race [21] and strain-dependent differences in oxygen induced ROP in the inbred rats [22-24]. Because three of the four FEVR genes are mutated in advanced ROP and all four FEVR genes are involved in Wnt signaling pathway, the above genetic explanation of ROP supports a role for the Wnt signaling pathway in the development of severe ROP that can be of a therapeutic value in the future. Because it is the same pathway that appears to be defective in both of these disorders (ROP and FEVR), their clinical similarities can be explained. Considering all the available data to date, it appears that NDP, FZD4 and LRP5 gene polymorphisms can account for about 10-12% of ROP and this prevalence may be correlated with ethnic differences. However, the NDP, FZD4 and LRP5 genes are not the major genes independently accounting for a significant portion of ROP patients. This suggests that mutations in other genes involved in retinal development, angiogenesis and Wnt signaling pathway could also be associated with severe ROP in a small proportion of patients.

## Association of other genes with ROP

The blinding complication of ROP is strongly associated with the development of retinal neovascularization. In normal instances, the vascularization of the human retina is largely complete by the 4^th ^month of gestation but peripheral retinal vascularization will not be in place until the fetus is near term. ROP pathogenesis occurs in two phases: the vascular attenuation phase (phase I) and the fibrovascular proliferative phase (phase II). In phase I, hyperoxia (because of supplemental oxygen) causes cessation of normal retinal vascularization and in phase II, hypoxia renews vascularization. In both of these cases vascular endothelial growth factor (VEGF) plays a major role [25,26] and depending on local retinal responses, the effect can be normal or abnormal vascularization. Several case control studies have also confirmed the association of VEGF single nucleotide polymorphisms (SNPs) with diseases as diverse as breast cancer, oral cancer, Alzheimer disease and kidney disease. It has also been reported that increased expression of VEGF gene is associated with both avascular retina and intravitreous neovascularization [27] in a model of ROP. Many polymorphisms of VEGF gene have been described. Some of them are in the promoter and 5'-untranslated region but some of them are in the 3'-untranslated region (3'-UTR). Several polymorphisms within the VEGF gene are correlated with variation in VEGF protein production [28]. For instance, it was reported previously [29] that the CC genotype of C936T polymorphism in the 3'-UTR of VEGF gene was associated with an increase in the VEGF level in the peripheral blood circulation as compared with CT and TT genotypes. In support of the VEGF gene involvement in ROP, some studies have shown an association of VEGF gene polymorphism and ROP [30,31] but these results are not replicated by other studies [32-36].

Additionally, it has also been reported that a prolonged period of low levels of insulin-like growth factor-I (IGF-I) may predict the development of ROP and other complications of premature birth [37]. Infants with higher IGF-I do not develop ROP and exhibit better vascular development. This growth factor (IGF-I) is an intrauterine growth factor and is expressed in retinal cells. Several studies also suggest that it is essential for vascular development of the eye in the postnatal period. These results were supported by IGF-I knockout mice that developed abnormal retinal vascular growth. Because prematurity is one of the factors that contributes to ROP and the IGF-I level is determined by the IGF-I receptor (IGF-I R) and the most prevalent polymorphism of IGF-I R (3174 G to A) exhibited low levels of free plasma IGF-I, it is possible that it may have a role in ROP. However, studies [38,39] do not support the association of this polymorphism and the risk of advanced ROP in different populations. Similarly, angiotensin-converting enzyme gene polymorphism is found to be associated with ROP in Kuwaiti population [16] but not in the other population [40,41]. Additionally, suggestive association has been reported between AGTR1 (encodes angiotensin II type I receptor), IHH (Indian Hedgehog), TBX-5 (T-box 5), glycoprotein Ib alpha polypeptide (GP1BA) and cholesterol ester transfer protein (CETP) and development of ROP [42]. However, these results need to be confirmed in a larger and independent population.

## Relationship between FEVR and advanced ROP

**Figure 2:**
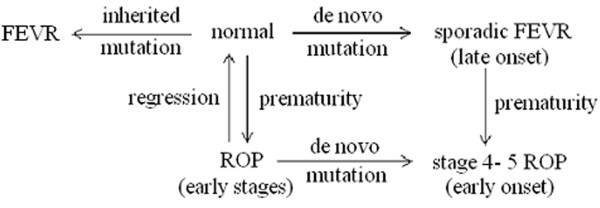
**A highly hypothetical pathway that may lead to a cicatricial ROP and a morphologically similar disorder FEVR**. *De novo *gene mutation coupled with environmental factors (e. g. prematurity) may lead to the progression and development of retinal detachment in stage 4 - 5 ROP whereas prematurity alone without gene mutation may lead to an early stage of ROP that may subsequently regress to a normal condition. On the other hand, inherited or *de novo *mutations without prematurity may result in familial and sporadic FEVR respectively, that develop during the first decade of life.

While it remains to be seen whether the recently reported fourth gene (TSPAN12) of FEVR is also involved in advanced ROP patients, the above studies demonstrated that mutations are present in either small percentage of ROP patients or limited geographically to a specific ethnic population. Although there are many questions such as accuracy of diagnosis and small percentage of ROP patients harboring mutations in FEVR genes that need to be addressed before any conclusion can be drawn, based on the studies conducted from around the world involving different ethnic background patients, it is possible that the advanced ROP could be in fact a sporadic FEVR and the prematurity is an environmental factor that simply speeds up the progression of the disease. Thus, it is conceivable that in ROP, gene abnormality coupled with postnatal physiological changes and various environmental factors (e.g. prematurity) may result in the development and progression of ROP that would ordinarily be suppressed in a full term infant with FEVR. Based on these assumptions we propose the following (Fig. 2), purely speculative, double-hit-working model to explain the development of advanced ROP in premature infants. In stage 4 - 5 ROP, *de novo *gene mutation coupled with environmental factors (e.g. prematurity) may lead to the progression and development of retinal detachment whereas prematurity alone without gene mutation may lead to an early stage of ROP that may subsequently regress to a normal condition. On the other hand, inherited or *de novo *mutations without prematurity may result in familial and sporadic FEVR respectively, which develop during the first decade of life.

## Wnt signaling pathway

**Figure 3:**
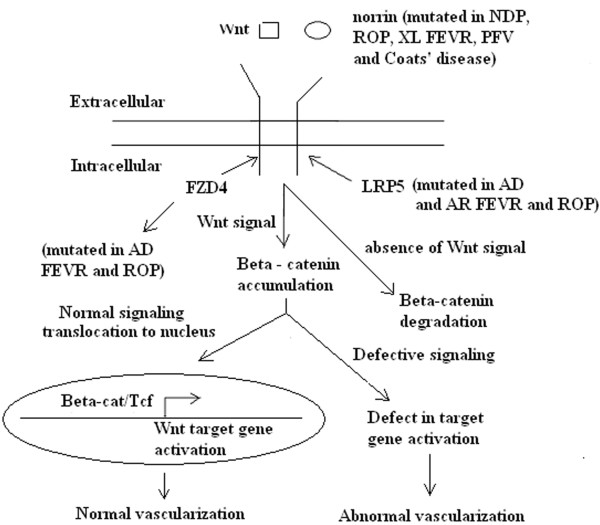
**A schematic representation of the canonical Wnt signaling pathway**. Norrin and Wnt act as ligands to bind FZD4 that interact with LRP5. In the absence of Wnt signaling, beta-catenin is phosphorylated and subjected to proteosomal degradation. In the presence of Wnt signaling, beta-catenin accumulates in the cytoplasm and enters the nucleus. Its subsequent interactions with a member of Tcf/Lef family activate the transcription of Wnt target genes. It was also shown that TSPAN12 is a component of the norrin-LRP5-FZD4 signaling complex and enhances the levels of norrin-beta-catenin signaling but not Wnt-beta-catenin signaling. Because of mutations in NDP, FZD4, LRP5 and TSPAN12 genes, abnormal signaling may occur which may result in defective Wnt target genes activation that may give rise to FEVR and ROP pathology. AD = autosomal dominant; AR = autosomal recessive; XL = X-linked recessive; FEVR = familial exudative vitreoretinopathy; ROP = retinopathy of prematurity; NDP = Norrie disease pseudoglioma.

The exact mechanism to explain how the mutant genes thus far identified cause incomplete vascularization of the retina in ROP is not known. However, mutations in the NDP, FZD4 and LRP5 genes in ROP and the finding that the same genes are also mutated in a morphologically similar disorder FEVR suggest that a similar mechanism may be involved in ROP and FEVR pathogenesis. Interestingly, as mentioned above, all the above four genes are involved in the Wnt signaling pathway. Therefore, the defective Wnt signaling pathway may be responsible for both FEVR and ROP pathogenesis. The Wnt signaling pathway (Fig. 3) is highly conserved and regulated among many species. It plays a key role in embryonic development including eye development. It was also shown that TSPAN12 is a component of the norrin -FZD4 - LRP5 - signaling complex and increases the levels of norrin-beta-catenin signaling but not Wnt-beta-catenin signaling. The protein TSPAN 12 is a member of tetraspanin family and facilitates the formation of multimolecular membrane complexes. A series of experiments demonstrated that TSPAN 12 is required for the multimerization of FZD4. Recently, it has been reported that [43] a transcription factor called sox 17 is upregulated by norrin-FZD4-LRP5 signaling. This factor plays an important role in inducing the angiogenic program for retinal vascularization. Therefore, loss of or insufficient norrin-FZD4-LRP5 signaling (due to mutations) may cause defective vascular growth and that may lead to retinal hypovascularization, which is the predominant feature of both FEVR and ROP. Although there is no direct correlation between genotype and phenotype, alterations in highly conserved amino acids may affect the structure of receptors (FZD4 and LRP5), ligand (NDP), localization of mRNA or proteins or the quantity of proteins. This may inactivate or alter the pathway resulting in the inhibition or abnormality of vascular development in the above disorders. Mutations in the 5' and 3'-untranslated regions (seen in NDP gene in ROP patients) may also alter the regulation of gene expression at the level of transcription, translation and mRNA stability. This may result in profound changes in the signal transduction (reduction in signaling) and hence produce changes in vascular development [44]. Mice lacking NDP, FZD4, TSPAN12 and LRP5 [45-48] further demonstrate the importance of wild-type genes in capillary maturation as well as this pathway in vasculogenesis and normal retinal development. However, these transgenic experiments must be interpreted with caution because, in many patients mutations in NDP, FZD4, LRP5 and TSPAN12 are missense whereas in mice models the above four genes are not expressed (null mutants). Further understanding of this pathway in ocular disease may lead to a novel therapeutic approach to treat or prevent these potentially blinding disorders in the future. However, large-scale studies involving different ethnic groups and direct experimental evidence to show that the same pathway is involved in ROP are needed to either confirm or refute the above suggestion.

## Conclusion

ROP is a leading cause of blindness in children. It is unknown why some extremely premature babies develop severe ROP despite timely intervention whereas other babies with similar clinical characteristics do not progress to a severe stage. From the foregoing evidence it is clear that genetic factors in addition to prematurity or environmental factors play a major role in the development and progression of ROP. Identification of polymorphisms or mutations in genes is only the beginning and it may not solve all the problems. We need to consider bioinformatic and proteomic approaches at a given point in time [24]. A comparison of protein profile between normal and affected individuals throughout the course of the disease may provide a better diagnostic indicator. For instance, the mutated gene may be under the control of environmental factors such as oxygen exposure or prematurity and may not be expressed, over expressed or change the expression pattern of other genes. The proteomics approach directly addresses the status of genes. There are several promising biomarkers for the risk of ROP. For example, deamination of globin chains appears to be a promising marker [49]. Additionally, it is possible that ROP involves multiple genes rather than a single gene, each gene then contributing a small but additive effect resulting in the final phenotype. Thus, along with genomics, bioinformatics and proteomics approaches may ultimately provide a better management of ROP.

## Competing interests

The author declares that they have no competing interests.

## Authors' contributions

The manuscript was prepared by BSS.
